# IMPACT OF NEIGHBORHOOD GREEN INFRASTRUCTURE ON ACCESS TO SOCIAL CAPITAL ACROSS THE LIFE COURSE

**DOI:** 10.1093/geroni/igac059.2302

**Published:** 2022-12-20

**Authors:** Noah Webster, Natalie Sampson, Joan Nassauer

**Affiliations:** University of Michigan, Ann Arbor, Michigan, United States; University of Michigan-Dearborn, Dearborn, Michigan, United States; University of Michigan, Ann Arbor, Michigan, United States

## Abstract

While the impact of neighborhood characteristics on access to social capital is well established, less is known about how neighborhood landscape design interventions play a role in shaping access to this resource and how this varies across the life course. In this study we examined the association between age and perceived impact of recently installed neighborhood block-scale green infrastructure (GI) on frequency of social interactions with neighbors. We also examined age variation in how alternative GI designs were perceived (e.g., how well cared for), and how these perceptions were associated with the anticipated impact on frequency of neighbor interactions. Data are from the Neighborhood, environment, and water research collaborations for green infrastructure (NEW-GI) project based in Detroit, MI. Four neighborhood GI interventions were installed in two Detroit neighborhoods in 2016. Surveys were conducted with residents living around the interventions in 2017-18 (n=171), and in nearby neighborhoods (n=145). Age was significantly associated with perceived impact of the landscape interventions on frequency of social interactions with neighbors. Specifically, older adults were significantly more likely to report that the landscape interventions that they were most familiar with resulted in increased frequency of interactions with their neighbors. Further, design alternatives perceived as more well cared for were anticipated to result in greater increases in the frequency of interactions with neighbors among older compared to younger adults. Results suggest neighborhood landscape interventions can improve access to social capital particularly among older adults, and perceptions of landscape care play a role in this process.

